# P003: Current status of infection control practice for prevent of central venous catheter-associated bloodstream infection in Korea

**DOI:** 10.1186/2047-2994-2-S1-P3

**Published:** 2013-06-20

**Authors:** PG Choe, HY Shin, MJ Shin, K-H Song, ES Kim, HY Jin, YH Choi, OJ Choi, KH Park, NJ Park, K-H Kim, SH Han, EJ Choo, HB Kim

**Affiliations:** 1 Seoul National University, Seoul, Korea, Republic Of; 2 Seoul National University Hosptial, Seoul, Korea, Republic Of; 3 Seoul National University Bundang Hospital, Seongnam, Korea, Republic Of; 4 Ajou University Hospital, Suwon, Korea, Republic Of; 5 Chonnan National University Hospital, Gwangju, Korea, Republic Of; 6 Pusan National University Hospital, Pusan, Korea, Republic Of; 7 Soon Cheon Hyang University Bucheon Hospital, Bucheon, Korea, Republic Of

## Introduction

There are evidence-based guidelines for the prevention of central line-associated bloodstream infections (CLA-BSI), but the current status of these practices in intensive care units (ICU) of Korea is unknown.

## Objectives

To evaluate the current status of infection control practice for CLA-BSI in ICUs of Korea.

## Methods

We conducted a cross-sectional survey in ICUs of the KOrean Study group for Infection Control and prevention (KOSIC) at April 2012.

## Results

Thirty-five ICUs of 15 hospitals were enrolled in this study. Fourteen of the 35 ICUs (40%) were medical ICUs, 4 (11%) were surgical ICUs, 9 (26%) were neurosurgical ICUs, and 8 (23%) were combined medical and surgical ICUs. The median bed size was 15 beds (interquartile range [IQR], 14-20), and median patient-to-nurse ratio was 1.5 (IQR, 1.3-1.9). During the survey period, the incident rate of CLA-BSI was 3.33 per 1,000 catheter-days (medical ICUs, 5.12; surgical & neurosurgical ICUs, 1.91; combined ICUs, 2.25).

All ICUs had documented guidelines for the prevention of CLA-BSI and conducted surveillance for CLA-BSI. Nineteen (54%) ICUs provided regular education programs for CLA-BSI prevention and 15 (43%) ICUs accessed the adherence to guidelines using a central line insertion checklist. Twenty-nine (83%) ICUs used a sterile full body drape during an insertion practice and 3 (8%) ICUs used chlorhexidine preparation with alcohol for an insertion skin preparation. Twenty (57%) ICUs used antimicrobial-impregnated coated central venous catheter.

All ICUs conducted hand hygiene promotion program including adherence monitoring and 23 (66%) ICUs conducted active surveillance for multidrug resistant organisms. Hand hygiene adherence was significantly associated with the patient to nurse ratio in ICU (γ = 0.648, P < 0.001).

## Conclusion

This study demonstrates that although ICUs in Korea had documented guideline and surveillance system for CLS-BSI, infection control practice in real clinic did not meet the recommended practice standard.

## Disclosure of interest

None declared

